# Apoptosis is mediated by FeHV-1 through the intrinsic pathway and interacts with the autophagic process

**DOI:** 10.1186/s12985-023-02267-w

**Published:** 2023-12-12

**Authors:** Gianmarco Ferrara, Consiglia Longobardi, Maria Francesca Sgadari, Brunella Restucci, Giuseppe Iovane, Roberto Ciarcia, Ugo Pagnini, Serena Montagnaro

**Affiliations:** Department of Veterinary Medicine and Animal Productions, University of Naples Federico II, Via Federico Delpino n.1, Naples, 80137 Italy

**Keywords:** Apoptosis, Feline herpesvirus, Caspases, Intrinsic pathway, Autophagy

## Abstract

**Background:**

Although FeHV-1 is a primary feline pathogen, little is known about its interactions with host cells. Its relationship with several cellular pathways has recently been described, whereas its interplay with the apoptotic process, unlike other herpesviruses, has not yet been clarified. The aim of this work was to evaluate whether FeHV-1 induces apoptosis in its permissive cells, as well as the pathway involved and the effects of induction and inhibition of apoptosis on viral replication.

**Methods:**

Monolayers of CRFK cells were infected at different times with different viral doses. A cytofluorimetric approach allowed the quantification of cells in early and late apoptosis. All infections and related controls were also subjected to Western blot analysis to assess the expression of apoptotic markers (caspase 3-8-9, Bcl-2, Bcl-xL, NF-κB). An inhibitor (Z-VAD-FMK) and an inducer (ionomycin) were used to evaluate the role of apoptosis in viral replication. Finally, the expression of autophagy markers during the apoptosis inhibition/induction and the expression of apoptosis markers during autophagy inhibition/induction were evaluated to highlight any crosstalk between the two pathways.

**Results:**

FeHV-1 triggered apoptosis in a time- and dose-dependent manner. Caspase 3 cleavage was evident 48 h after infection, indicating the completeness of the process at this stage. While caspase 8 was not involved, caspase 9 cleavage started 24 h post-infection. The expression of other mitochondrial damage markers also changed, suggesting that apoptosis was induced via the intrinsic pathway. NF- κB was up-regulated at 12 h, followed by a gradual decrease in levels up to 72 h. The effects of apoptosis inhibitors and inducers on viral replication and autophagy were also investigated. Inhibition of caspases resulted in an increase in viral glycoprotein expression, higher titers, and enhanced autophagy, whereas induction of apoptosis resulted in a decrease in viral protein expression, lower viral titer, and attenuated autophagy. On the other hand, the induction of autophagy reduced the cleavage of caspase 3.

**Conclusions:**

In this study, we established how FeHV-1 induces the apoptotic process, contributing to the understanding of the relationship between FeHV-1 and this pathway.

**Supplementary Information:**

The online version contains supplementary material available at 10.1186/s12985-023-02267-w.

## Introduction

Programmed cell death (PCD) plays a key role in both physiological (such as development or immune regulation) and pathological events (such as neurodegeneration, cancer and infectious diseases) [1–3]. Apoptosis, one of the best-studied PCDs, is considered a critical process during normal development and host defense mechanisms for eliminating cells via a complex but well-defined program [4]. The process can be divided into two phases: the initiation phase, which depends on the nature of the stimuli, and the central pathway (effector phase), which is common to all apoptotic processes and involves a family of aspartate-specific-cysteinyl-proteases (caspases) activated by proteolytic cleavage [2, 3]. The initiation of apoptosis can occur via the extrinsic or intrinsic pathway [4]. The first involves the activation of the death domain receptors (such as Fas, TNF-related apoptosis-inducing ligand receptors 1 and 2) activated by a series of proteins (as FasL ligand, TRAIL, TNFm). Following these interactions, oligomerization occurs and the death-inducing signaling complex (DISC) is formed. FADD, recruited during a specific cascade, plays a key role in this process and activates the extrinsic apoptosis initiator caspase 8, which subsequently activates effector caspases (3,6 and 7). The second is activated by proapoptotic stimuli, causing permeabilization of mitochondrial membrane, which allows intermembrane proteins, such as cytochrome c, to be released into the cytosol [5, 6]. This sequence of events triggers the intrinsic apoptosis initiator caspase-9 as well as downstream effector caspases of the extrinsic route [5]. The B-cell lymphoma 2 (Bcl-2) protein family regulates the intrinsic pathway at the mitochondrial level. Several Bcl-2 family proteins are antiapoptotic, such as Bcl-2, whereas others, such as Bcl-2-associated X protein (BAX), Bcl-2 antagonist or killer (BAK), and Bcl-2 homology domain (BH3)-interacting domain death agonist (BID), are proapoptotic [7]. Antiapoptotic proteins block the proapoptotic proteins BAX and BAK by sequestering BH3-only proteins like BID. Nuclear fragmentation and cell morphological changes (membrane blebbing, cell shrinkage, and chromatin condensation leading to apoptotic bodies formation) and nuclei are representative outcomes of apoptosis and are widely used as markers for apoptosis [2]. The executioner caspase-3, whose activation creates the morphological markers of apoptosis, is addressed through both extrinsic and intrinsic routes. During viral infection in animals, apoptosis promotes lysis of infected cells (preventing or reducing virus multiplication in other cells) and allows phagocytosis of dying cells by macrophages (converging in the immune and inflammatory responses) [8]. Although apoptosis contributes to the prevention of viral pathogenesis (by limiting viral replication and transmission), it has been defined as a “potentially costly and even vainly attempted self-sacrifice” [3, 9]. On the other hand, viruses have acquired anti-apoptotic strategies to escape apoptotic pathways and maintain their replication and propagation [1]. Several viruses complete the replication before the onset of apoptosis (rapid proliferation). Others prevent apoptosis by targeting and regulating key regulatory steps and then prolonging the cell survival of infected cells thanks to antiapoptotic proteins [9]. Several anti-apoptotic proteins are encoded by viruses, including US3, gJ, and LAT expressed by HSV-1, CrmA of cowpox virus, p35 and IAPs of baculovirus, Bcl-2, encoded by either Epstein-Barr virus, African swine fever virus, or Herpesvirus saimiri [7, 9–11]. Moreover, apoptosis inhibition is essential for the maintenance of latent infection in several herpesviruses [12]. In comparison to other herpesviruses, little is known about the relationship between FeHV-1, the etiological agent of feline rhinotracheitis, and apoptosis. A previous study has demonstrated how the PI3K/Akt/mTOR axis is altered during FeHV-1 infection [13]. However, it has not been defined where the activation of this pathway converges in the apoptotic process, although this pathway plays a role in several cellular processes. This study aimed to learn more about this relationship by evaluating the impact of apoptosis on FeHV-1 infection in permissive cells.

## Methods

### Cell culture, Infection and annexin V-FITC assay

Crandell-Rees Feline Kidney Cells (CRFK) cultured in Dulbecco’s Modified Eagle’s Medium (DMEM; Corning) were infected using MOI 1 of FeHV-1 strain Ba/91 at different time points (3, 6, 12, 24, 48, and 72 h) to reveal the time dependence activation of apoptosis during FeHV-1 infection (cell characteristics were observed using the light microscopy ZOE Cell Imager, Bio-Rad Laboratories). Similarly, CRFK cells were infected using increasing MOIs of FeHV-1 (0.1-1-10) and UV-inactivated virus for 48 h. Infected and control cells were collected using trypsin, centrifuged, washed with PBS 1X, and resuspended in Annexin binding buffer (Cell Signaling). A total of 96 µl of cell resuspension (approximately 10,000 cells) was mixed with 12.5 µl propidium iodide, 1 µl Annexin V-FITC conjugate and incubated on ice, in the dark for 10 min. An annexin binding buffer was filled to a final volume of 250 µl. Cells stained with PI and Annexin V-FITC (late apoptotic cells) or Annexin V-FITC only (early apoptotic) were visualized and counted using a FACS (BD FACSCalibur, BD Bio-sciences). A total of 5,000 events were recovered, and data were analyzed using BD cellQuest Pro software version 3.3 (BD Biosciences, San Jose, CA, USA). Flow cytometric analysis was carried out as previously described [14].

### Western blot assay

Pellets derived from the previously described infections were lysed with RIPA buffer containing protease and phosphatase inhibitors (Sigma), quantified by Braford assay (BioRad), and eluted with Laemmli 5x. Sodium Dodecyl Sulphate - PolyAcrylamide Gel Electrophoresis (SDS-page) was performed using pre-casted gels (BioRad) transferred to nitrocellulose membranes (BioRad). After a blocking step carried out in 5% bovine serum albumin (BSA), each membrane was incubated for one hour with a primary antibody eluted 1:1000 in BSA. The following antibodies were used: Caspase 3 (Cell Signaling), Caspase 9 (Cell Signaling), Caspase 8 (Santa Cruz Technologies), Bcl-2 (Abcam), Bcl-XL (Cell Signaling), NF-κB (Cell Signaling), β-Tubulin (Cell Signaling), β-Actin (Santa Cruz Technologies). After three wash steps, membranes were incubated with a secondary antibody (Anti-rabbit IgG, HRP-linked Antibody or Anti-mouse IgG, HRP-linked Antibody, Cell Signaling). Clarity Western ECL Substrate (Bio-rad) and a ChemiDoc Blot scanner (Bio-Rad) were used for visualization. Image Lab software was used to detect protein bands and assess expression levels using densitometric analysis (BioRad).

### Chemical induction and inhibition of apoptosis: evaluation of autophagy markers

The effects of apoptosis induction and inhibition on FeHV-1 were evaluated by Western blot, flow cytometry and TCID_50_. Briefly, cells were incubated overnight with Z-VAD-FMK (25 µm) and ionomycin (20 µm) before being infected with FeHV-1 for 48 h. Cell pellets were processed using Western blot (using specific FeHV-1 and caspase-3 antibodies) and flow cytometry analysis as previously described. Viral titers were determined using the TCID_50_ of the supernatants following the Reed-Muench method. The viability of control and treated cells was assessed by the MTT assay as described in previous studies [15]. Protein lysates from cell pellets were run on SDS-page as previously described, transferred to Polyvinylidene Difluoride (PVDF) membranes, and incubated with anti-rabbit LC3-I/II and SQSTM1/p62 antibodies (Cell Signaling). Incubation with secondary antibodies and visualization were performed as previously specified.

### Chemical induction and inhibition of autophagy: evaluation of apoptosis markers

To investigate the modifications that induction and inhibition of autophagy have on the FeHV-1-induced apoptotic process, CRFK monolayers were treated with bafilomycin and rapamycin before infection, as previously described [15]. After 48 h, pellets were collected and used for Western blot analysis, evaluating the expression of caspase 3 and cleavage.

### Statistical analysis

Three independent replicates were performed for each experiment. GraphPad Prism 6.0 (GraphPad Software, Inc., La Jolla, CA, USA) was used to perform one-way ANOVA, which were expressed as the mean standard deviation (SD). A p value < of 0.05, 0.01, and 0.001 was considered statistically significant and graphically represented with one, two, and three asterisks, respectively.

## Results

### FeHV-1 induces apoptosis in a time-dependent manner

**Fig. 1:**
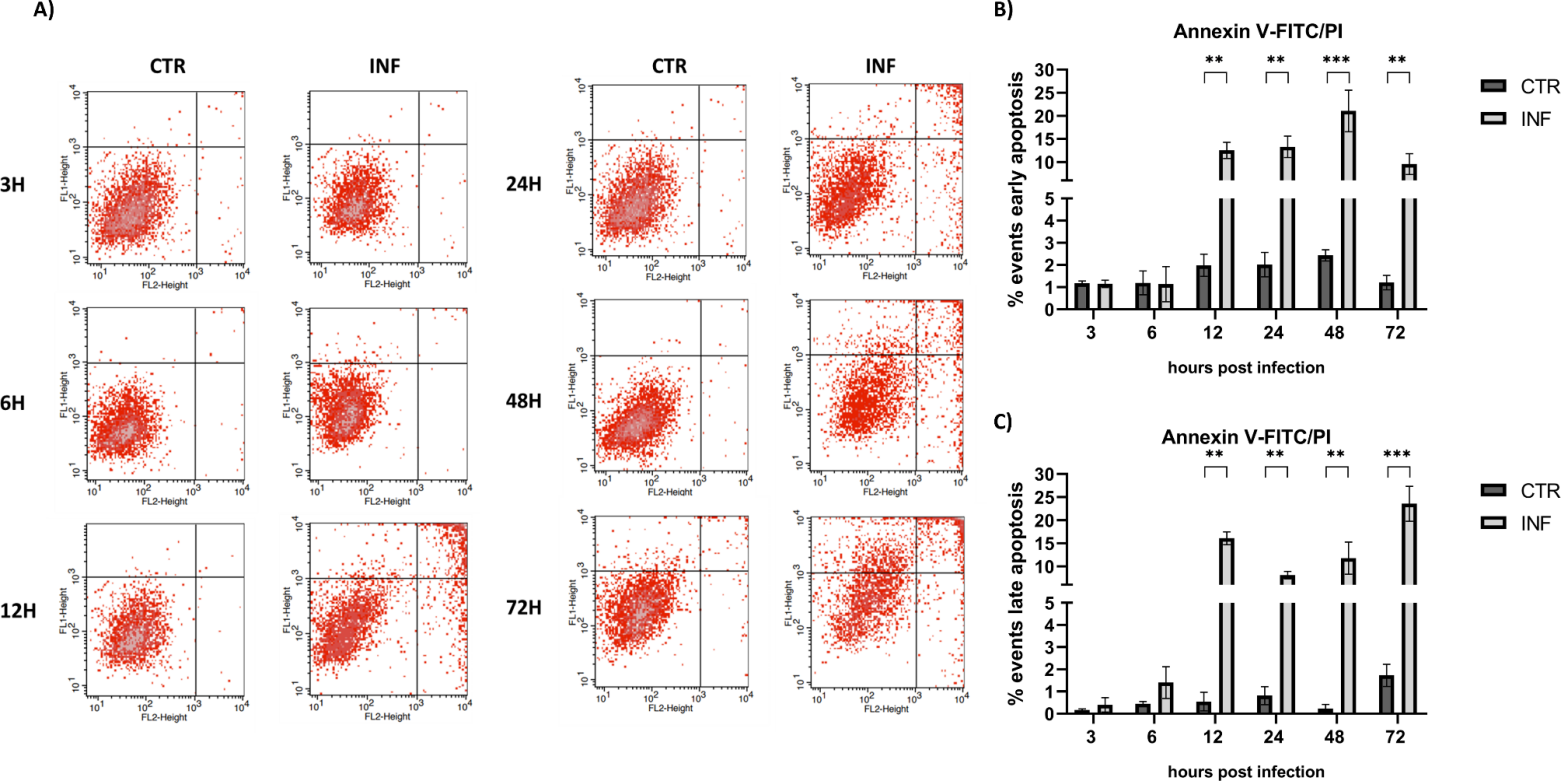
Flow cytometric analysis of FeHV-1 induced apoptosis at different time points. Flow cytometric pattern of control and infected cells at different times **(A)** and quantization of cells in early and late apoptosis (**B** and **C**)

FACS analysis quantified live, apoptotic, and dead cells following FeHV-1 infection. FeHV-1 triggered considerably apoptosis in a time-dependent manner, according to the results of cell staining with Annexin V-FITC and PI. At 12 h post infection, there was a substantial increase in both early and late apoptotic events (Fig. 1). The proportion of apoptotic cells increased with incubation time, peaking at 48 h after infection for early apoptosis events and 72 h after infection for late apoptotic events.

**Fig. 2:**
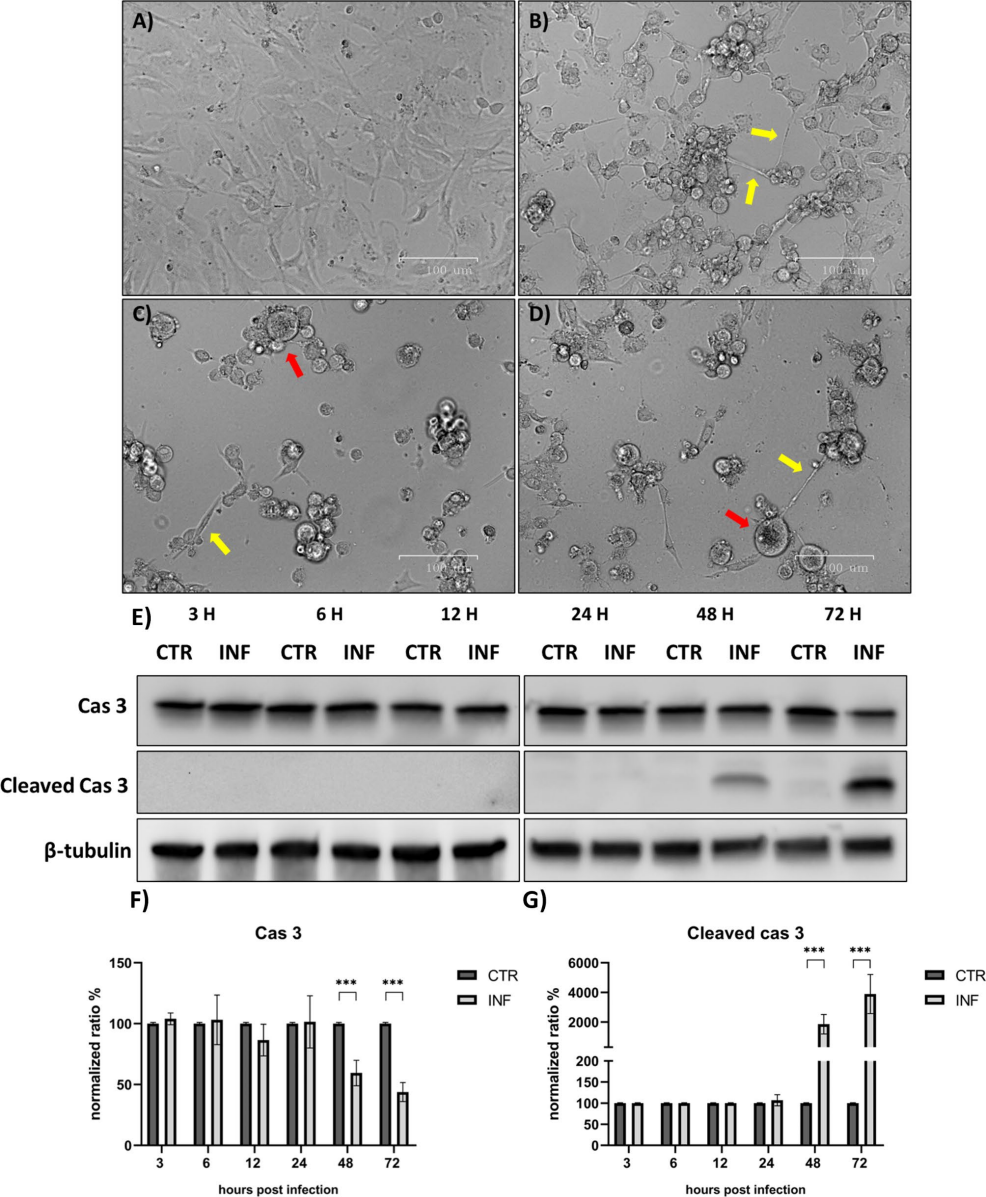
FeHV-1 induces apoptosis in a time-dependent manner. FeHV-1 triggers apoptosis in permissive CRFK cells. Images indicated with **B**, **C** and **D** showed how the cells appears after 24, 48 and 72 h from the infection (**A** indicated the control cells). The red arrows indicate the typical blebbing of apoptotic cells while the yellow arrows indicate the formation of pseudopods which connect the few cells that survived after the infection. The letter E indicates the expression of caspase 3 and its cleaved form at the various times of infection. The letters F and G indicated quantization after normalization. Full blots are available as supplementary file

**Fig. 3:**
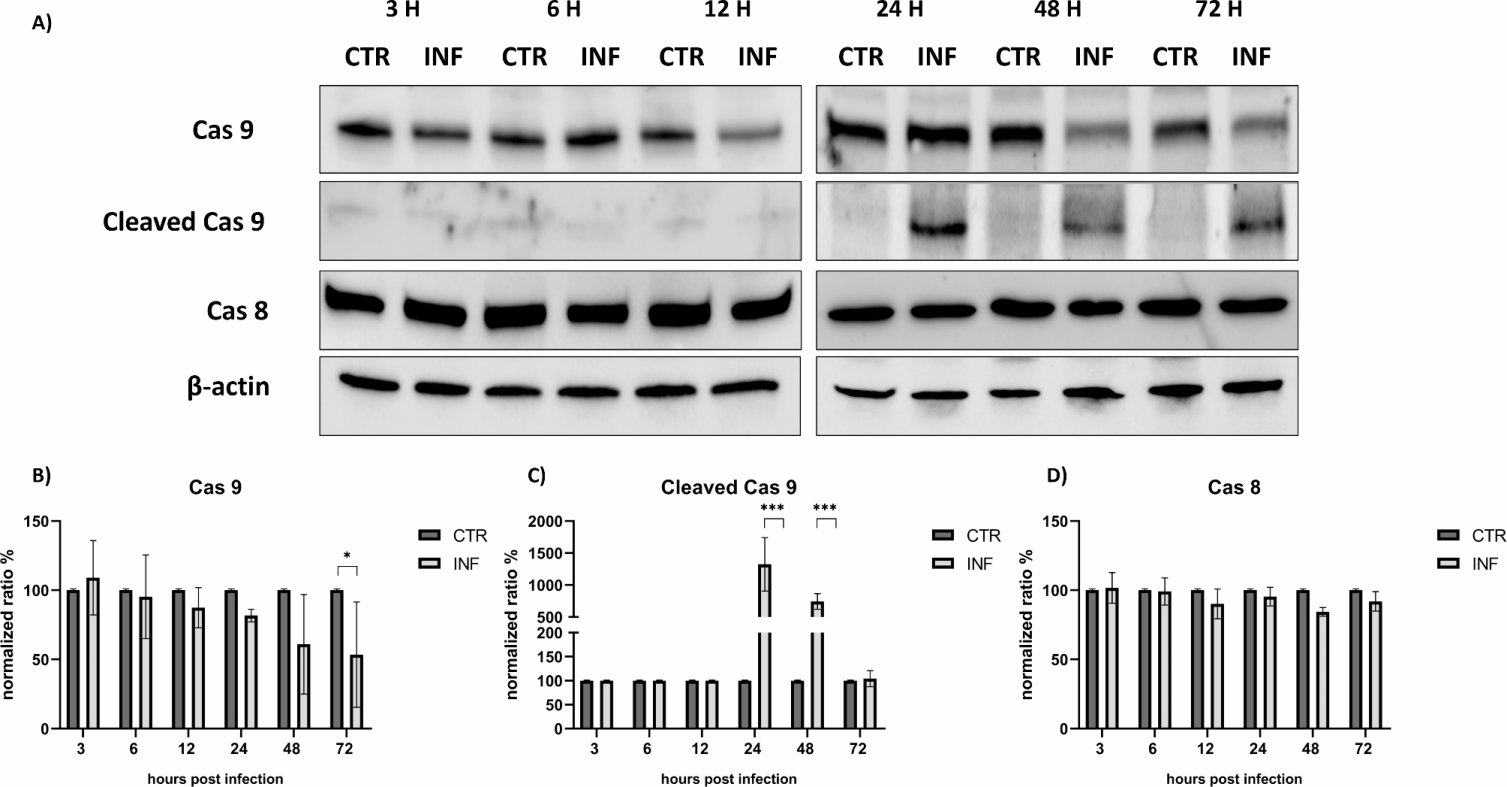
FeHV-1 induces caspase 9-cleavage after 24 h of infection. Western blot analysis of caspase 8, 9 and its cleaved form at different times of infection **(A)**. Indicated with the letters **B**, **C**, **D** are the expressions of the proteins after normalization. Full blots are available as supplementary file

Western blot analysis of caspase 3 expression and its cleaved form suggested that the apoptotic cascade caused by FeHV-1 occurred 48 h after infection. Cleavage of caspase-3 was evident at 48 h and became pronounced 72 h after infection (Fig. 2). At both time points described above, the costitutive form of caspase 3 decreased significantly. In Fig. 2 the phenotypic variations that cells infected with FeHV-1 undergo at different times were represented. The involvement of caspases 8 and 9 was also investigated and no significant changes over time for caspase 8 were observed, while caspase 9 was cleaved starting at 24 h post-infection (a decrease in constitutive form of caspase at 48 and 72 h after infection was observed) (Fig. 3).

**Fig. 4:**
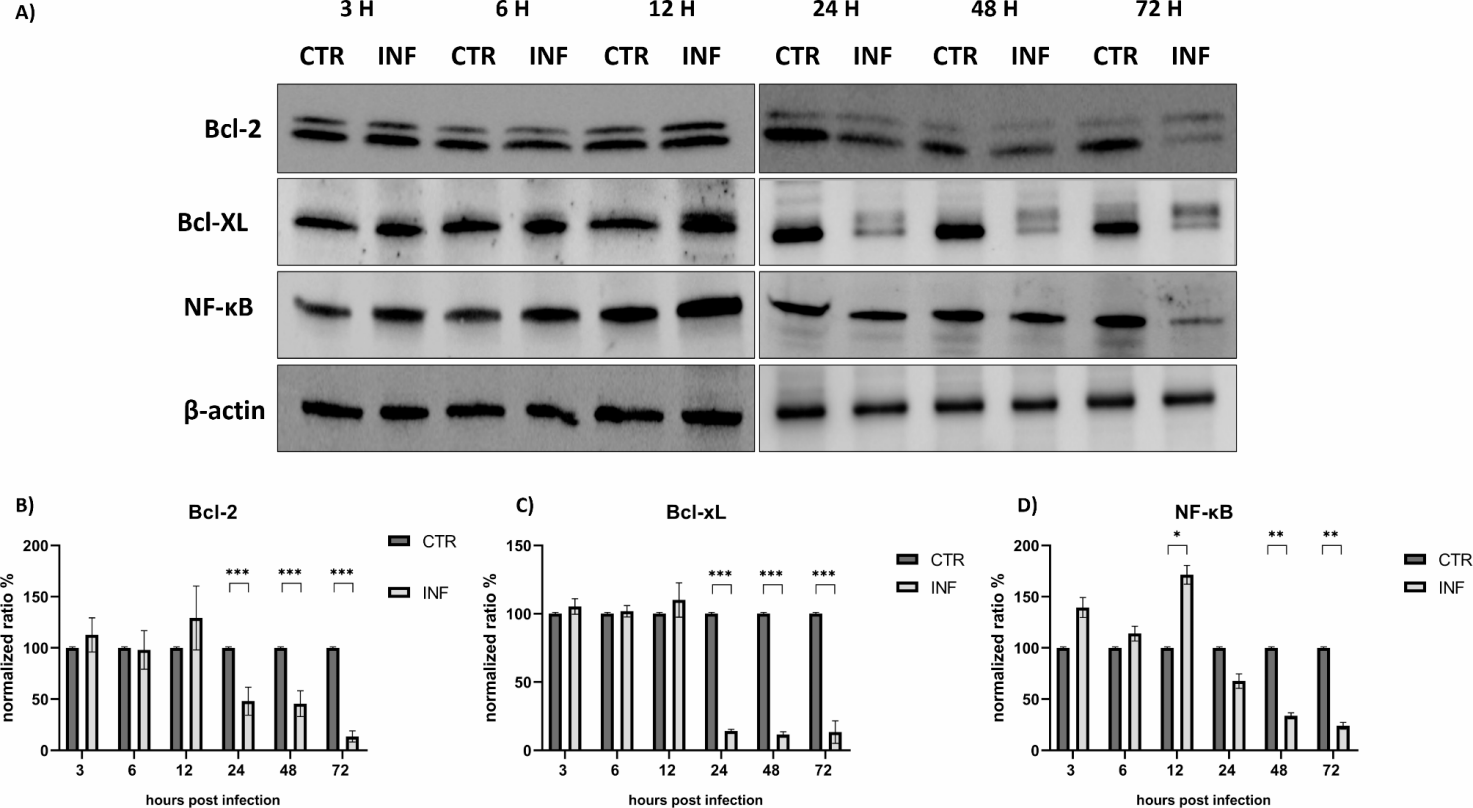
FeHV-1 perturbs the expression of Bcl-2, Bcl-xL and NF-κB. Western blot analysis of Bcl-2, Bcl-xL and NF-κB at different times of infection **(A)**. Indicated with the letters **B**, **C**, **D** are the expressions of the proteins after normalization. Full blots are available as supplementary file.

Given the intrinsic pathway’s role in the FeHV-1-induced apoptosis process, different markers of mitochondrial damage were investigated. In particular, western blot analysis showed a reduction of Bcl-2 and BcL-XL proteins starting 24 h post-infection, confirming the pronounced mitochondrial damage exhibited by FeHV-1-infected cells (Fig. 4). Evaluation of NF-κB expression revealed up-regulation at 12 h and subsequently a progressive reduction of the levels of this protein up to 72 h (Fig. 4).

### Relationship between apoptosis and viral dose during FeHV-1 Infection

**Fig. 5:**
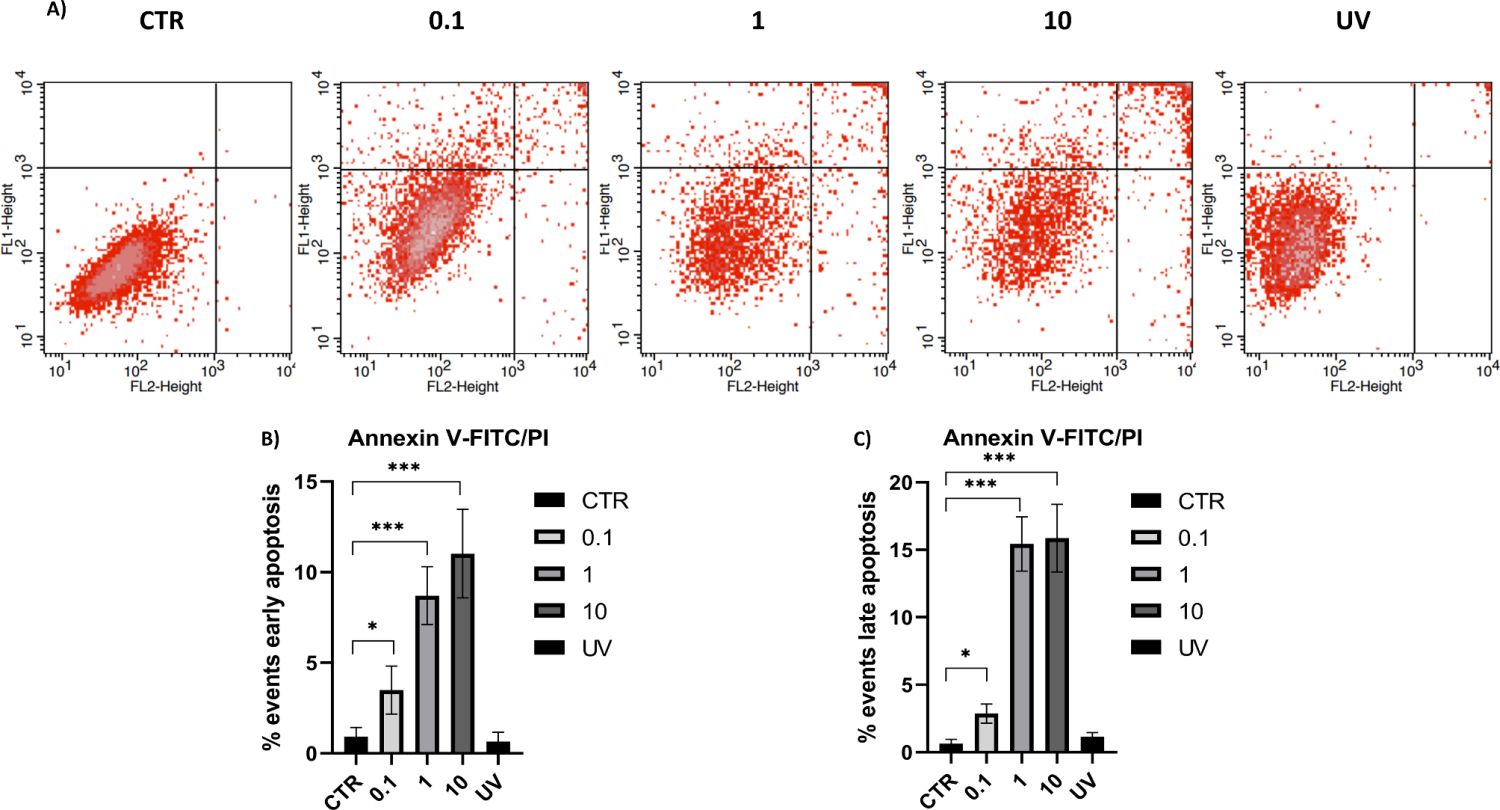
Flow cytometric analysis of FeHV-1 induced apoptosis at different MOIs. Flow cytometric pattern of control and infected cells at different MOIs **(A)** and quantization of cells in early and late apoptosis (**B** and **C**)

**Fig. 6:**
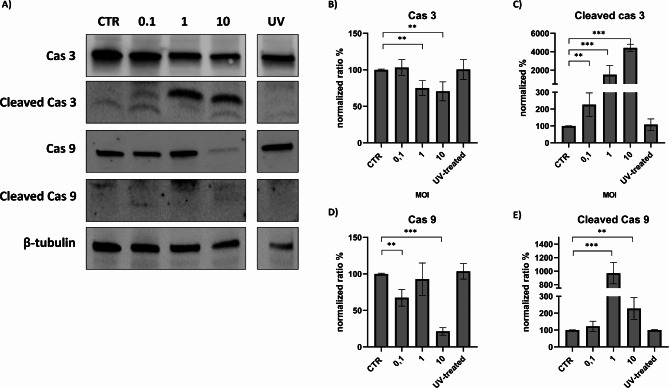
Changes in caspase 3 and 9 expression following FeHV-1 infection at different MOIs. Western blot analysis of caspase 3, 9 and its cleaved form at different MOIs **(A)**. Indicated with the letters **B**, **C**, **D**, **E** are the expressions of the proteins after normalization. Full blots are available as supplementary file

**Fig. 7:**
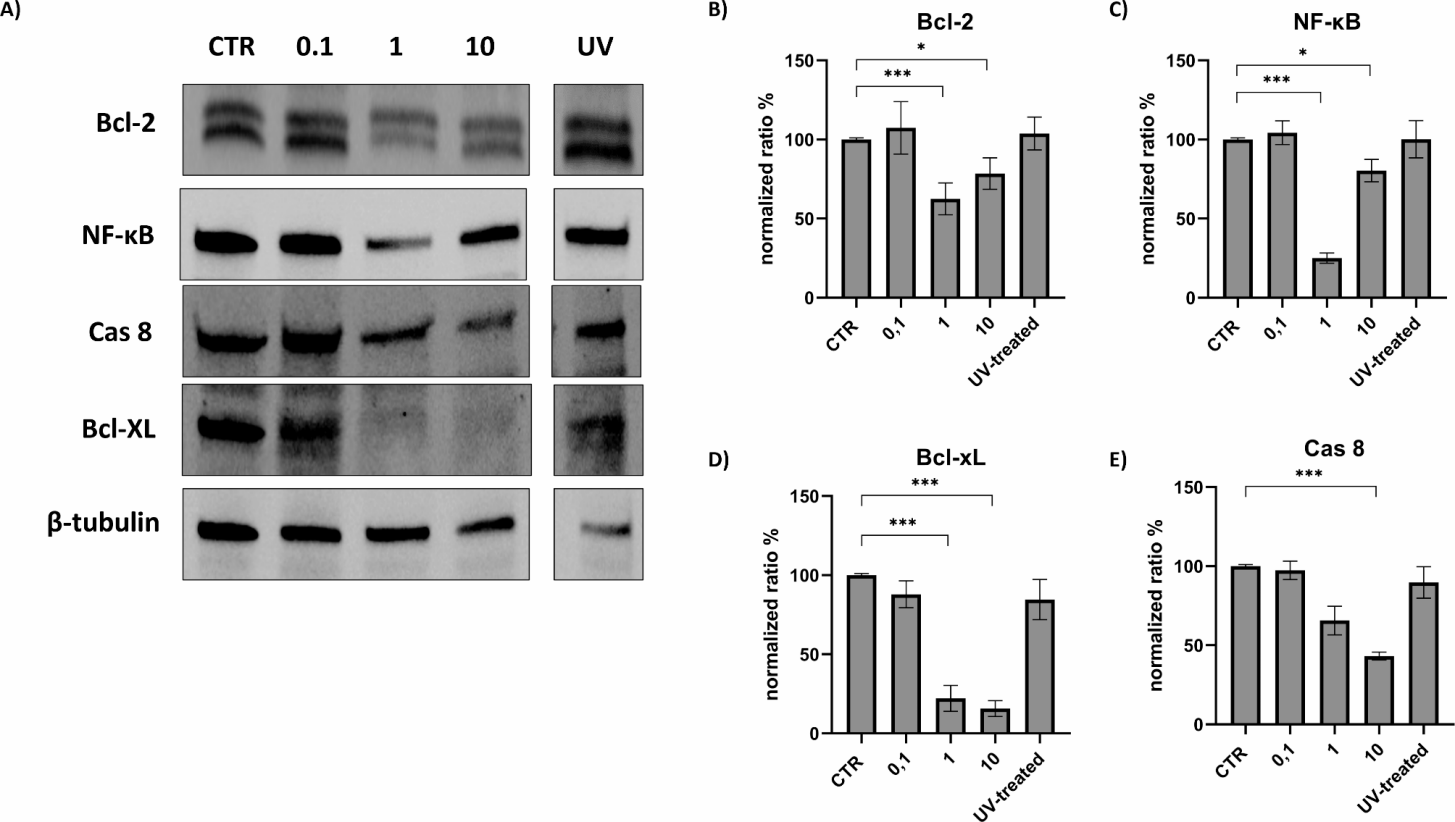
Changes in Bcl-2, Bcl-xL and NF-κB expression following FeHV-1 infection at different MOI. Western blot analysis of Bcl-2, Bcl-xL, caspase 8 and NF-κB at different MOIs **(A)**. Indicated with the letters **B**, **C**, **D**, **E** are the expressions of the proteins after normalization. Full blots are available as supplementary file

The parameters evaluated over time using MOI 1 were also evaluated using different MOIs at 48 h. Flow cytometric analysis showed a significant difference between MOI 0.1 and MOI 1, while the difference between MOI 1 and 10 appears to be negligible (Fig. 5). This pattern was confirmed when apoptotic markers were evaluated, with a substantial increase in caspase 9 and 3 cleavage between MOI 0.1 and MOI 1 (Fig. 6). The use of MOI 10 had no effect on caspase 3 cleavage compared to MOI 1 (Fig. 6). However, there was a considerable decrease in caspase 9, that was not coupled with an increase in the cleaved form. Similar patterns were observed for other markers such as Bcl-2, BcL-XL and NF-κB. The use of UV-inactivated virus did not cause any variation in terms of cell viability or expression of apoptosis markers (Fig. 7).

### Chemical induction and inhibition of apoptosis and implications for autophagy

**Fig. 8:**
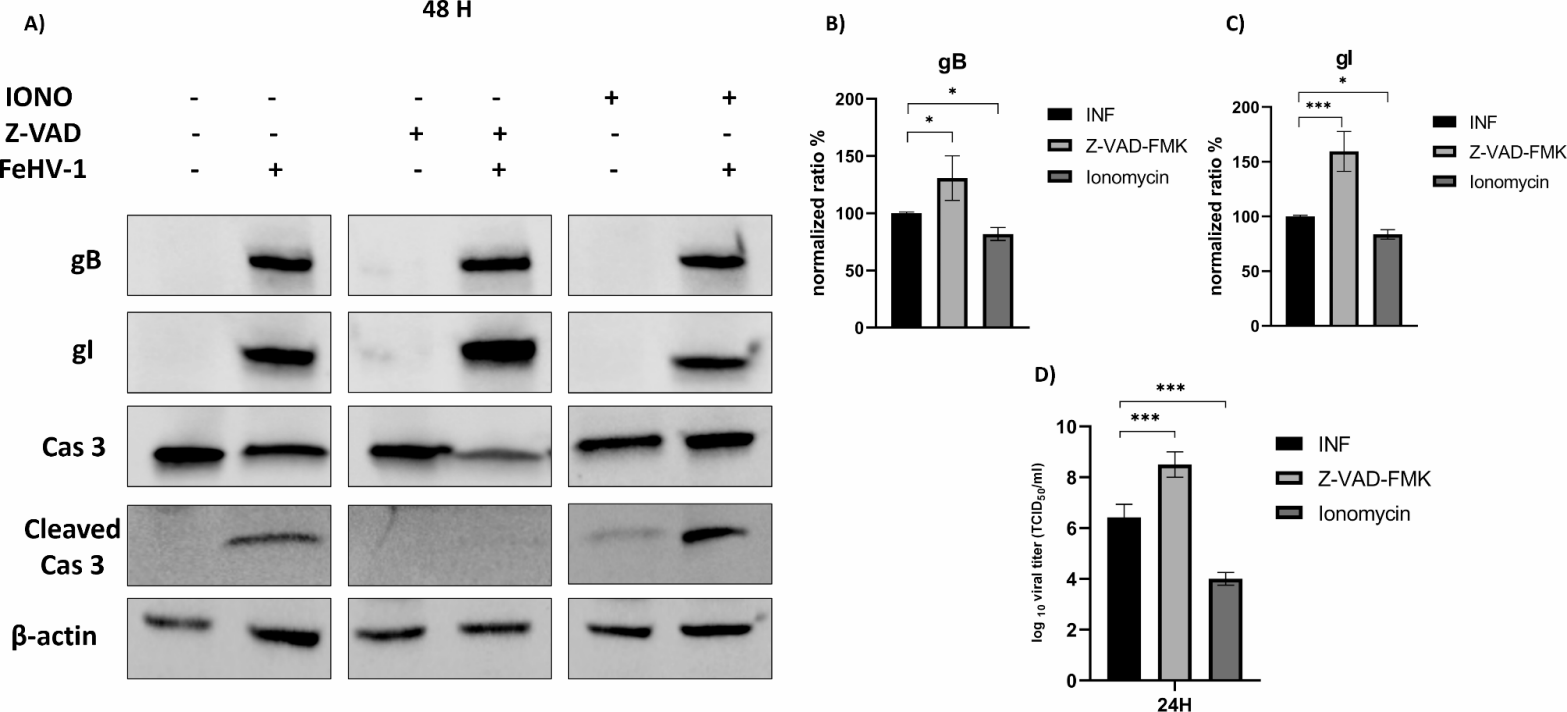
Effects of induction/inhibition of apoptosis on FeHV-1 replication. Western blot analysis of gB, gI, caspase 3 and its cleaved form during chemical induction/inhibition of apoptosis **(A)**. Indicated with the letters **B**, **C** are the expressions of the proteins after normalization. Indicated with the letter **D**, are viral titers (TCID50) obtained in supernatant of infected cells after induction/inhibition of apoptosis. Full blots are available as supplementary file

Subsequently, the effects of inhibitors and inducers of apoptosis on viral replication and apoptotic pathway were evaluated. The use of ZVAD, a pancaspase inhibitor, resulted in the prevention of caspase 3 cleavage, higher expression of viral glycoproteins, and higher viral titers (Fig. 8). Conversely, using ionomycin, an inducer of apoptosis, we obtained an increase in caspase 3 cleavage associated with a reduction in viral protein expression and viral titer (Fig. 8).

**Fig. 9:**
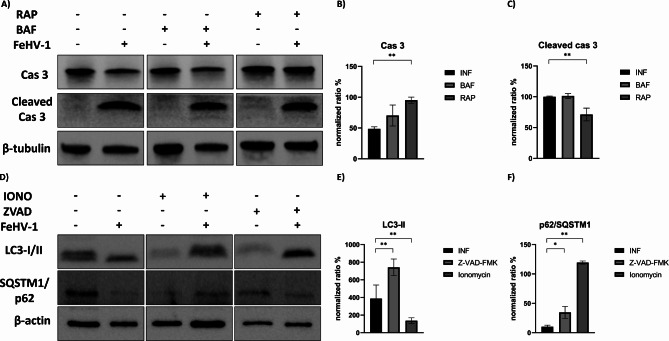
Crosstalk between apoptosis and autophagy during FeHV-1 infection. Western blot analysis of caspase 3 and its cleaved form after autophagy induction/inhibition **(A)**. Indicated with **B** and **C** are the expression of the proteins after normalization. Western blot analysis of LC3 and SQSTM1/p62 form after autophagy induction/inhibition **(D)**. Indicated with **E** and **F** are the expression of the proteins after normalization. Full blots are available as supplementary file

Furthermore, the two compounds seemed to have an opposite effect on FeHV-1-induced autophagy. In fact, while ionomycin attenuated the induction of autophagy caused by FeHV-1, ZVAD potentiated it (Fig. 9). Finally, caspase 3 was evaluated as an apoptotic marker during FeHV-1 infection in combination with an autophagy inhibitor (bafilomycin) and an inducer (rapamycin). Inhibition of autophagy did not cause any modification of caspase 3 cleavage, while rapamycin slightly reduced cleavage and increased the constitutive form (Fig. 9).

## Discussion

In this work, apoptosis induced by FeHV-1 in permissive cells was defined for the first time, obtaining important information that could be exploited in future antiviral strategies against this virus. Induction of apoptosis has been reported previously in several DNA and RNA viruses, including herpesviruses genomically related to FeHV-1. PRV, a member of the Varicellovirus family, modulates the expression of proapoptotic proteins (including Bcl-2) and cleavage of caspase 3 in a dose- and time-dependent manner [16]. Another example is BHV-1, which is able to induce apoptosis via the mitochondrial pathway in both MDBK-permissive cells and in neuronal cells (Neuro-2 A) [17, 18]. BHV-1 causes activation of caspase 9 and modulates the levels of Bcl-2 and Bax similarly to FeHV-1. Caprine herpesvirus type 1 (CpHV-1) induces apoptosis in both permissive and tumor cells by cleaving caspases 8 and 9 and damaging mitochondrial [19–21]. During Ovine herpesvirus 2 (OvHV-1) infection, mitochondria have been identified as the main target of viral-induced apoptosis, and the protein responsible for mitochondrial damage was also identified [22]. Viruses belonging to the same family can display a very different relationship with cellular apoptosis. In fact, many human herpesviruses encode proteins with antiapoptotic activity (Bcl-2 like proteins) with the aim of inhibiting or completely blocking the apoptosis pathway [9]. For example, HSV-1 has acquired Bcl-2 sequence, functional, and structural homologs in order to interfere with host death and autophagy signaling to promote its own replication [7, 10]. The link between herpesviruses and apoptosis varies depending on the type of infection. Apoptosis, in fact, represents a danger to some herpesviruses during latent infection, and some have acquired the ability to inhibit this process during latency. For example, Varicella Zoester virus (VZV), displays different outcomes according to the infected cell type, as it induces apoptosis in fibroblastic cells while making neuronal cells resistant to apoptosis [12, 23, 24]. All these events should be interpreted as an evasion of the wide range of host defenses (including the adaptive immune response), intended as a survival strategy for viruses during infection. Given that the virus inactivated with UV did not cause changes in the apoptotic process as measured by flow cytometry and western blot, it is likely that the apoptosis trigger is attributable to the expression of a viral gene or protein rather than a receptor type. In other alpha herpesviruses genomically related to FeHV-1 (such as VZV or PRV), the induction or inhibition of apoptosis occurs through specific proteins [1, 9]. For example, Us3 overexpression upregulates several anti-apoptotic genes, while UL16 triggers apoptosis. The same occurs during VZV infection in T cells through the ORF66 kinase [1, 9, 12].

For all previously described herpesviruses, at least 24 h are required to observe signs of programmed death in infected cells. In the case of FeHV-1, cleavage of caspase 3 is highlighted only after 48 h. It remains unclear whether this phenomenon appears at this moment because it was previously inhibited. It would be interesting to investigate whether the virus expresses some type of anti-apoptotic protein during this period that would allow it to prevent premature host cell death and conclude its replicative cycle during the first 24–48 h of infection.

Other viruses (both DNA and RNA) of veterinary interest have been described as inducers of the process of programmed cell death. Canine parvovirus (CPV) induces apoptosis via both intrinsic and extrinsic pathways in MDCK cells, while in Hela cells it does so via mitochondrial damage and via the NS1 protein [25, 26]. Other examples have been described for Canine coronavirus (CCV), Bluetongue virus (BTV), African horse sickness virus (AHSV), Porcine hemagglutinating encephalomyelitis virus (PHEV), porcine delta coronavirus (PDCoV), Porcine parvovirus (PPV), Porcine epidemic diarrhea virus (PEDV) [27–32].

In our study, we observed how pharmacological induction and inhibition of apoptosis had effects on viral replication as well as on apoptotic markers. The trend of viral titer and viral protein expression under apoptosis inhibitor treatment would imply that this mechanism has an antiviral effect. However, given the complex interaction between viruses and apoptosis, it was difficult to definitively establish the role of these processes. Due to its ability to eliminate cells compromised by viral infections, apoptosis is essential for host defense [1]. Apoptosis can be actively induced by viruses, reducing host immunological and inflammatory responses while allowing viral spreading to adjacent cells [5]. In other viruses, this correlation between the use of caspase inhibitors and reduction of the viral titer has been described, such as PDCoV and CPV [25, 31]. In other viruses, such as PHEV, the use of Z-VAD-FMK did not influence virus production [32].

The influence that infections have on the expression of NF-κB also varies between viruses [33]. This pathway is known to integrate signaling belonging to different intra- and extracellular stimuli (including viral infections) and elicit a proinflammatory response [34]. To replicate and persist within their hosts, viruses have evolved several strategies to evade and exploit cellular NF-κB immune signaling cascades for their benefit [33, 34]. In a recent study, FeHV-1 caused inhibition of NF-κB expression 8 h post-infection (at MOI 10) [35]. In general, the trend observed for FeHV-1, characterized first by up-regulation and then by down-regulation, is also described for herpesviruses related to FeHV-1, such as PRV [36, 37]. VZV also inhibits the NF-κB pathway [38].

In this study, we also found a crosstalk between autophagy and apoptosis induced by FeHV-1. Both mechanisms maintain homeostasis in cells and play essential roles in viral infections. In the case of FeHV-1, we demonstrated that apoptosis has an inverse effect on autophagy. Conversely, induction of autophagy has no effect on apoptosis, while the induction of autophagy reduces the extent of the process. This relationship has also been described in other viruses, and particularly PRV (autophagy induction inhibits apoptosis) [39]. Further studies are needed in order to highlight further links between the various cellular pathways for this prevalent feline virus [40]. According to what is present in the literature, it would seem that FeHV-1 uses the PI3K/Akt/mTOR axis mainly for its entry (already 30 min post infection), then induces autophagy (at 12 h post infection), mitochondrial damage (at 24 h), and complete apoptosis (at 48 h) [13, 15]. The presence of common markers suggests that these cellular processes should be considered as part of the same continuum rather than completely separate processes.

## Conclusion

In this study, we observed the induction of apoptosis mediated by FeHV-1 (in a dose- and time-dependent manner) and triggered by the intrinsic pathway and therefore by mitochondrial damage. The use of an apoptosis inducer resulted in a decrease in viral titer, and this could be properly exploited for new targets for antiviral drugs. We also evaluated the crosstalk between apoptosis and autophagy during FeHV-1 infection. All this information, which still need to be confirmed in vivo, enriches the knowledge of virus-host cell interactions in the context of FeHV-1.

### Electronic supplementary material

Below is the link to the electronic supplementary material.

Supplementary Material 1: Whole western blot membranes of figures 2, 3, 4, 6, 7, 8, and 9

## Data Availability

Data sharing not applicable to this article as no datasets were generated or analysed during the current study.
