# Supplementary material for: Apoptosis is mediated by FeHV-1 through the intrinsic pathway and interacts with the autophagic process
Source: Virol J. 2023 Dec 12;20:295. doi: 10.1186/s12985-023-02267-w (PMC10716993; doi:10.1186/s12985-023-02267-w)

FIG.2

CASPASE 3 AND CLEAVED CASPASE 3

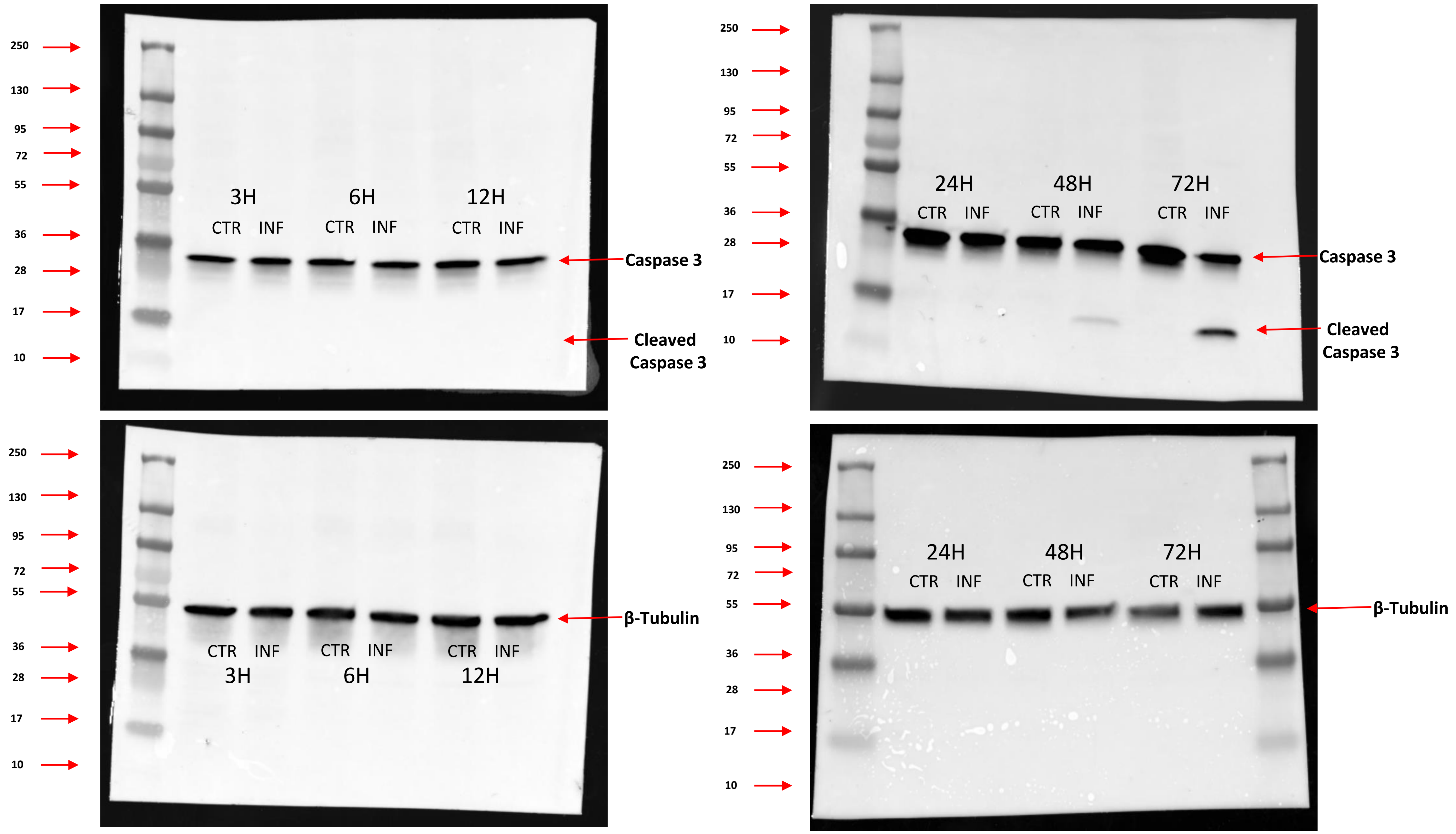

FIG.3

CASPASE 9 AND CLEAVED CASPASE 9

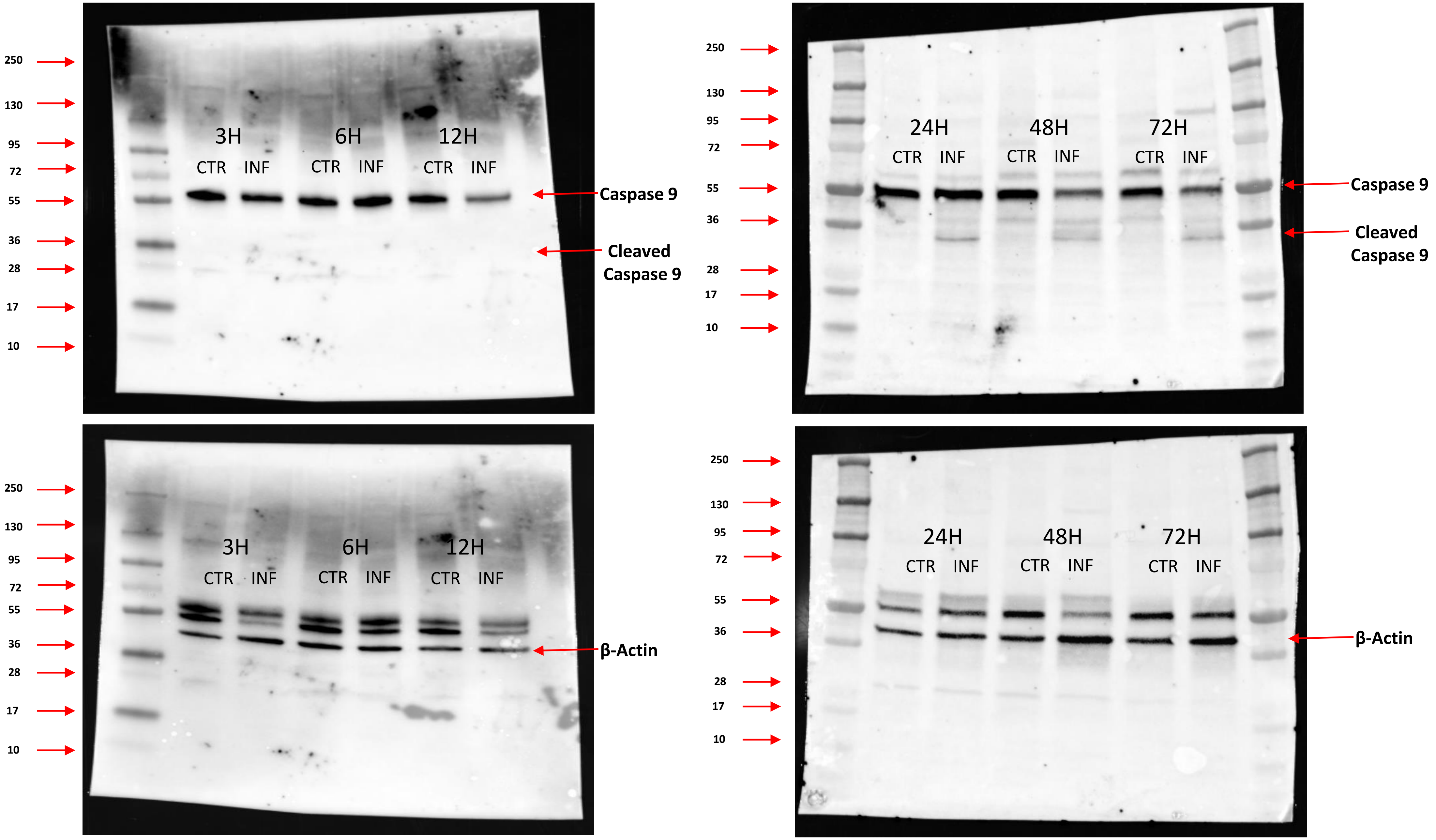

FIG.3

CASPASE 8 AND CLEAVED CASPASE 8

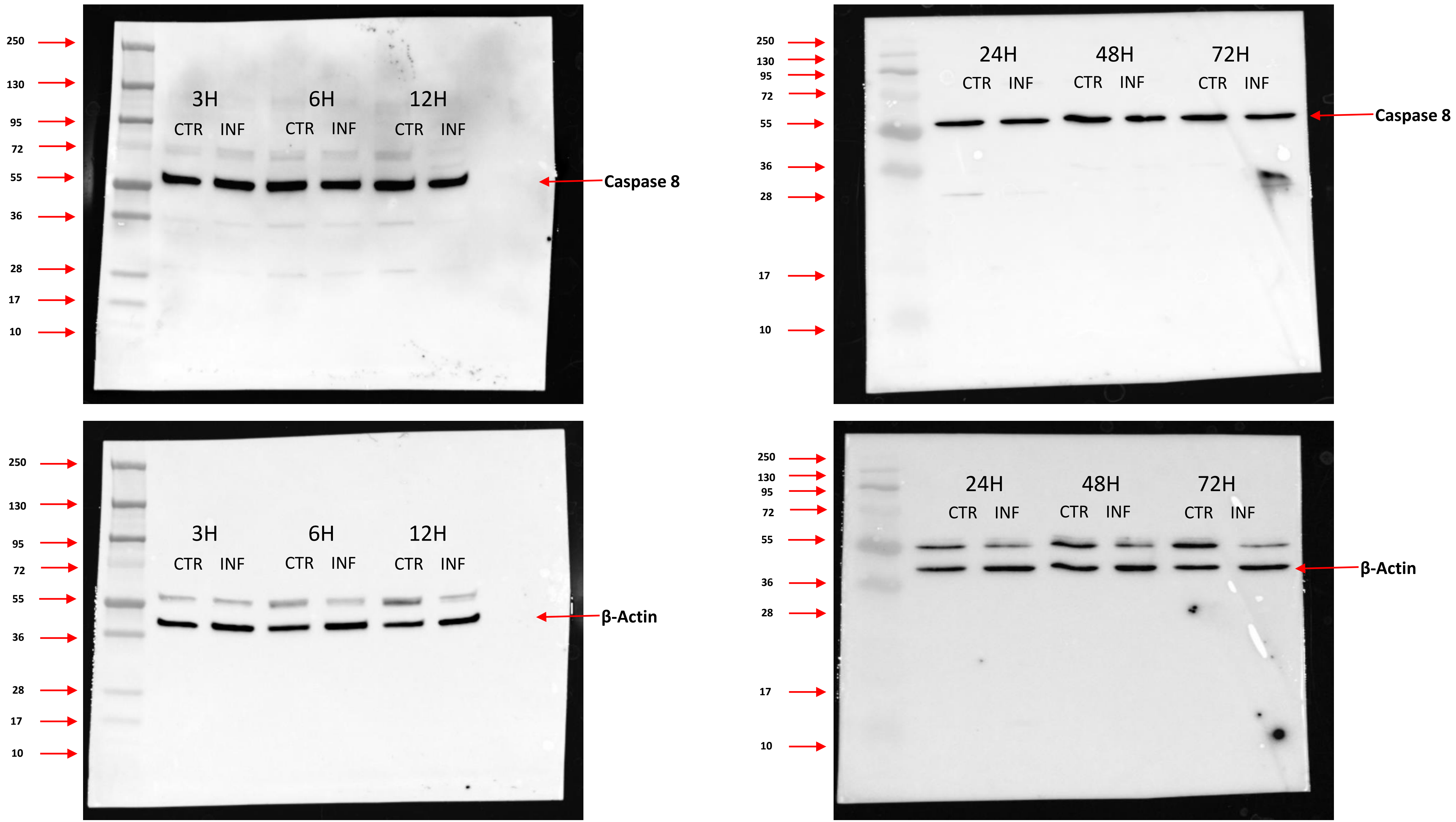

FIG.4

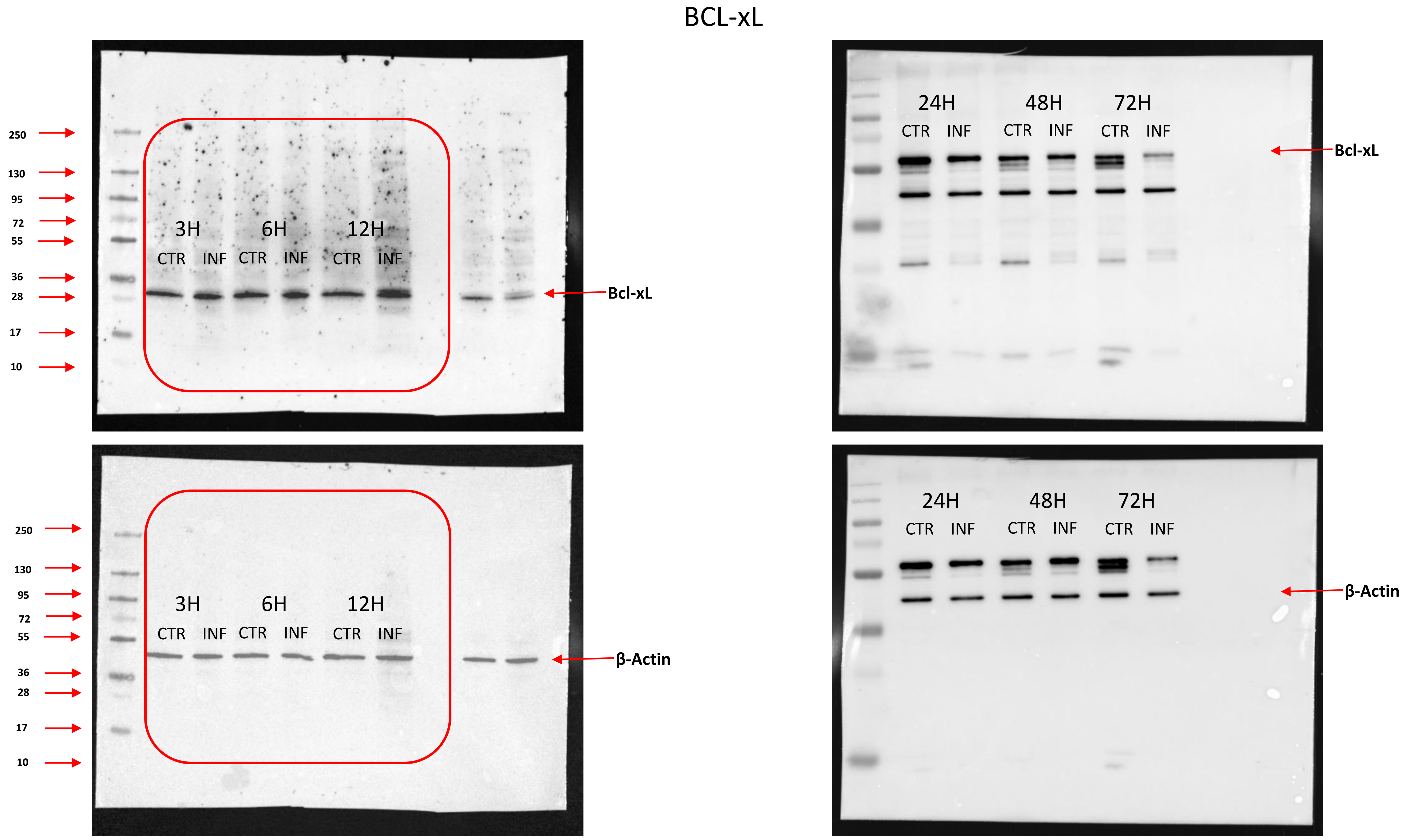
BCL-xL
250
130
95
72
55
36
28
17
10
3H
6H
12H
CTR
INF
CTR
INF
CTR
INF
Bcl-xL
24H
48H
72H
CTR
INF
CTR
INF
CTR
INF
Bcl-xL
250
130
95
72
55
36
28
17
10
3H
6H
12H
CTR
INF
CTR
INF
CTR
INF
β-Actin
24H
48H
72H
CTR
INF
CTR
INF
CTR
INF
β-Actin

FIG.4

BCL-2

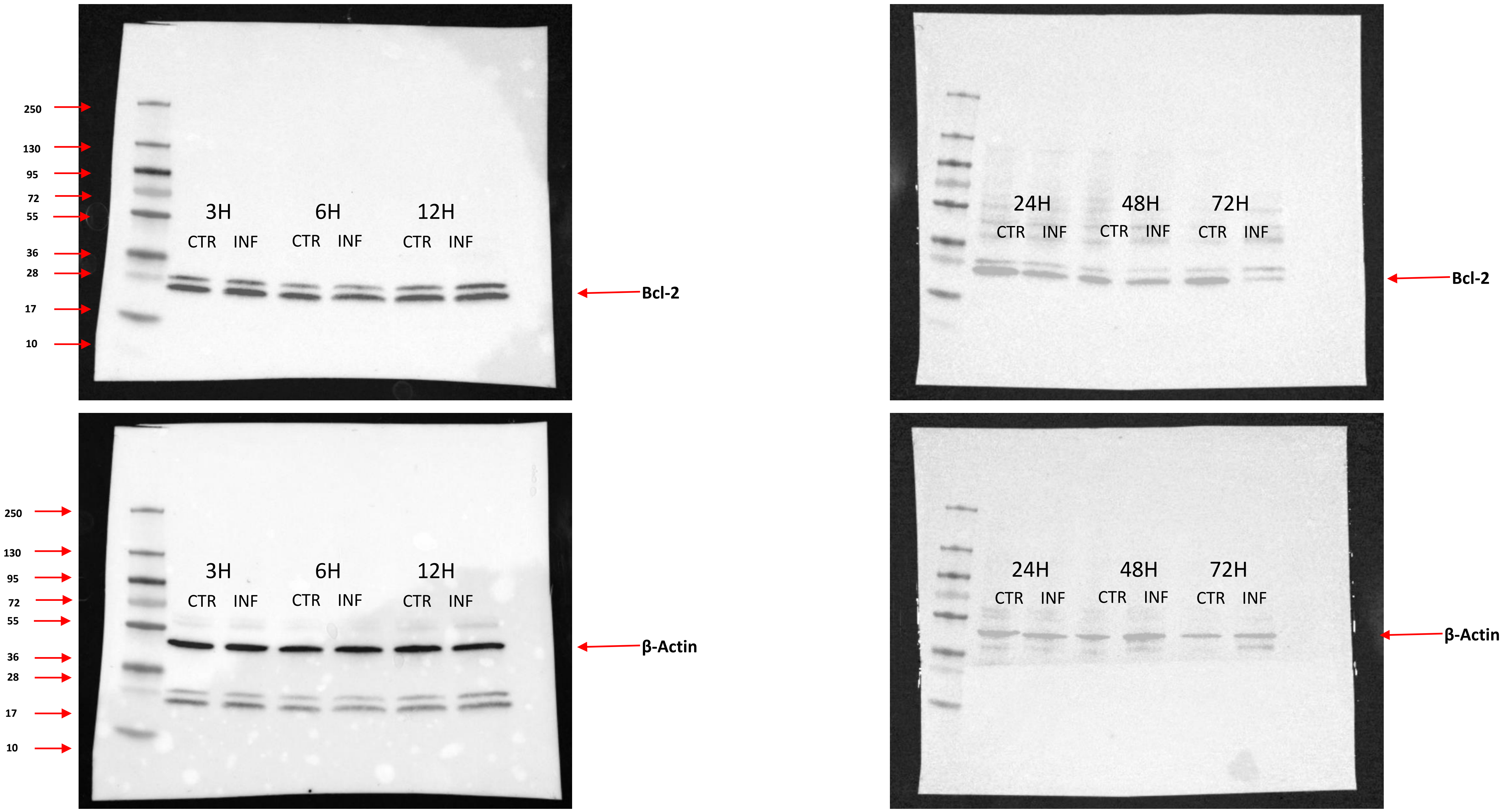
250
130
95
72
55
36
28
17
10
3H
6H
12H
CTR
INF
CTR
INF
CTR
INF
Bcl-2
24H
48H
72H
CTR
INF
CTR
INF
CTR
INF
Bcl-2
250
130
95
72
55
36
28
17
10
3H
6H
12H
CTR
INF
CTR
INF
CTR
INF
β-Actin
24H
48H
72H
CTR
INF
CTR
INF
CTR
INF
β-Actin

FIG.4

NF-kB

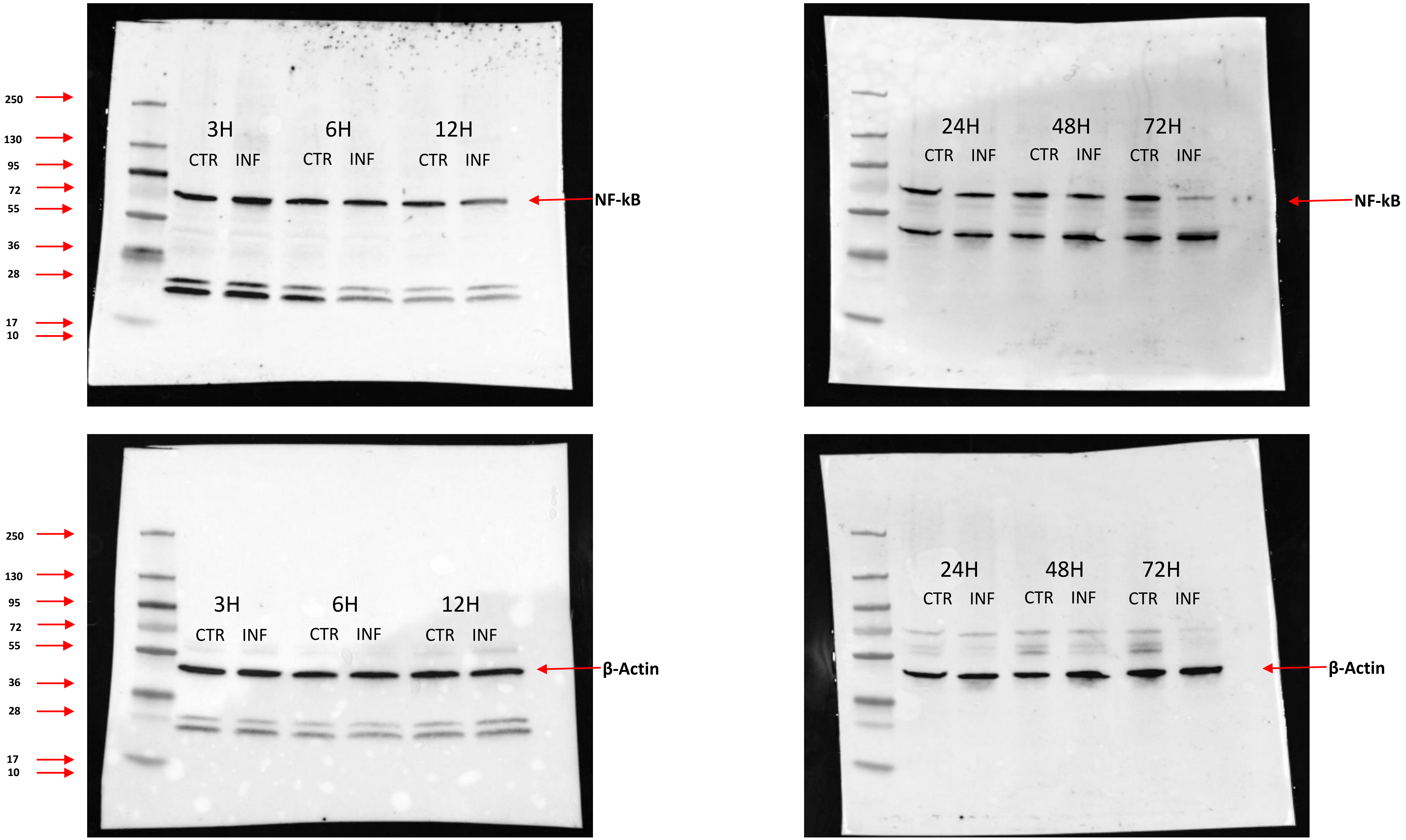
250
130
95
72
55
36
28
17
10
3H
6H
12H
CTR INF CTR INF CTR INF
NF-kB
24H
48H
72H
CTR INF CTR INF CTR INF
NF-kB
250
130
95
72
55
36
28
17
10
3H
6H
12H
CTR INF CTR INF CTR INF
β-Actin
24H
48H
72H
CTR INF CTR INF CTR INF
β-Actin

FIG.6-7

CASPASE 3, NF-kB AND BCL-2

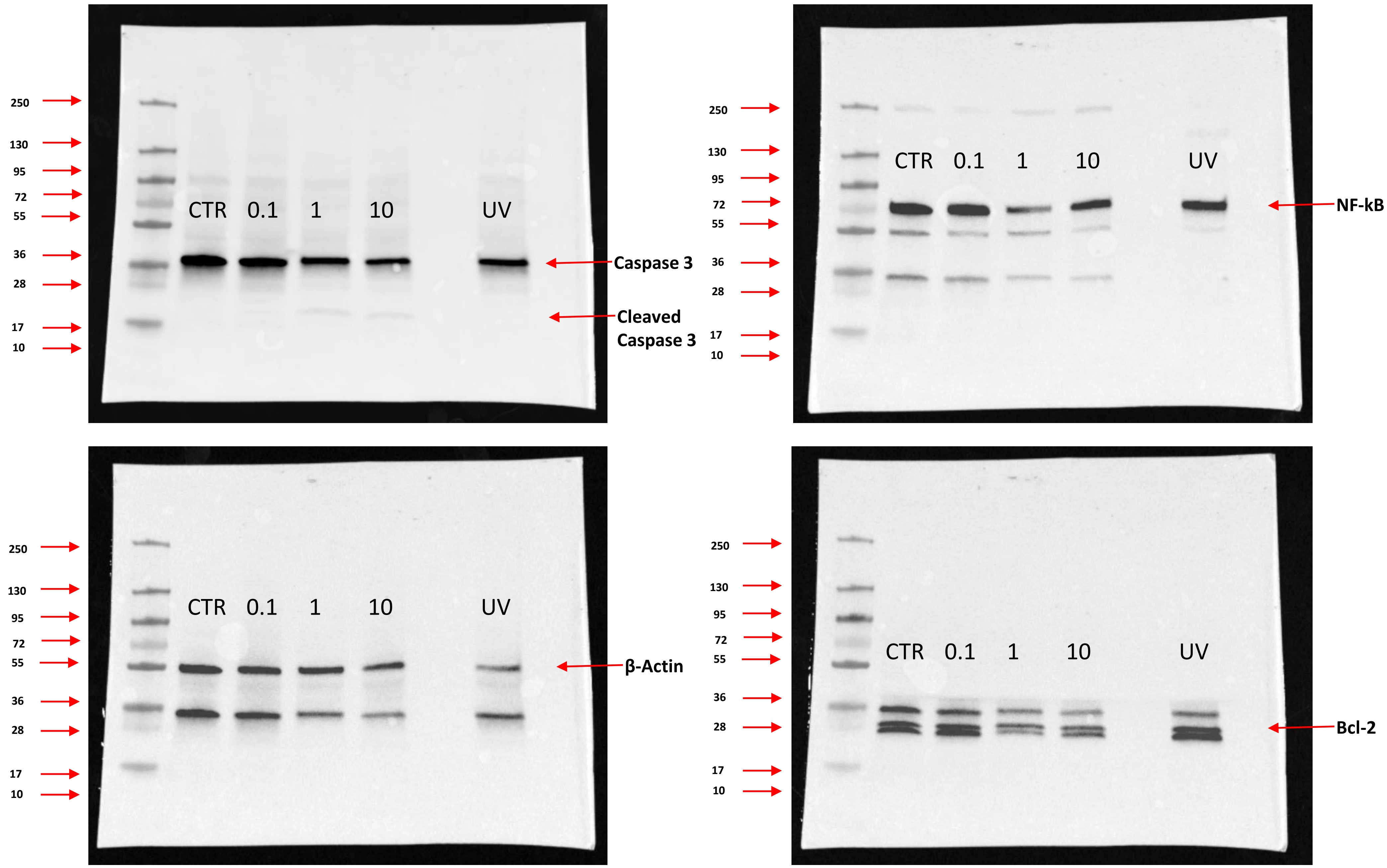

FIG.6

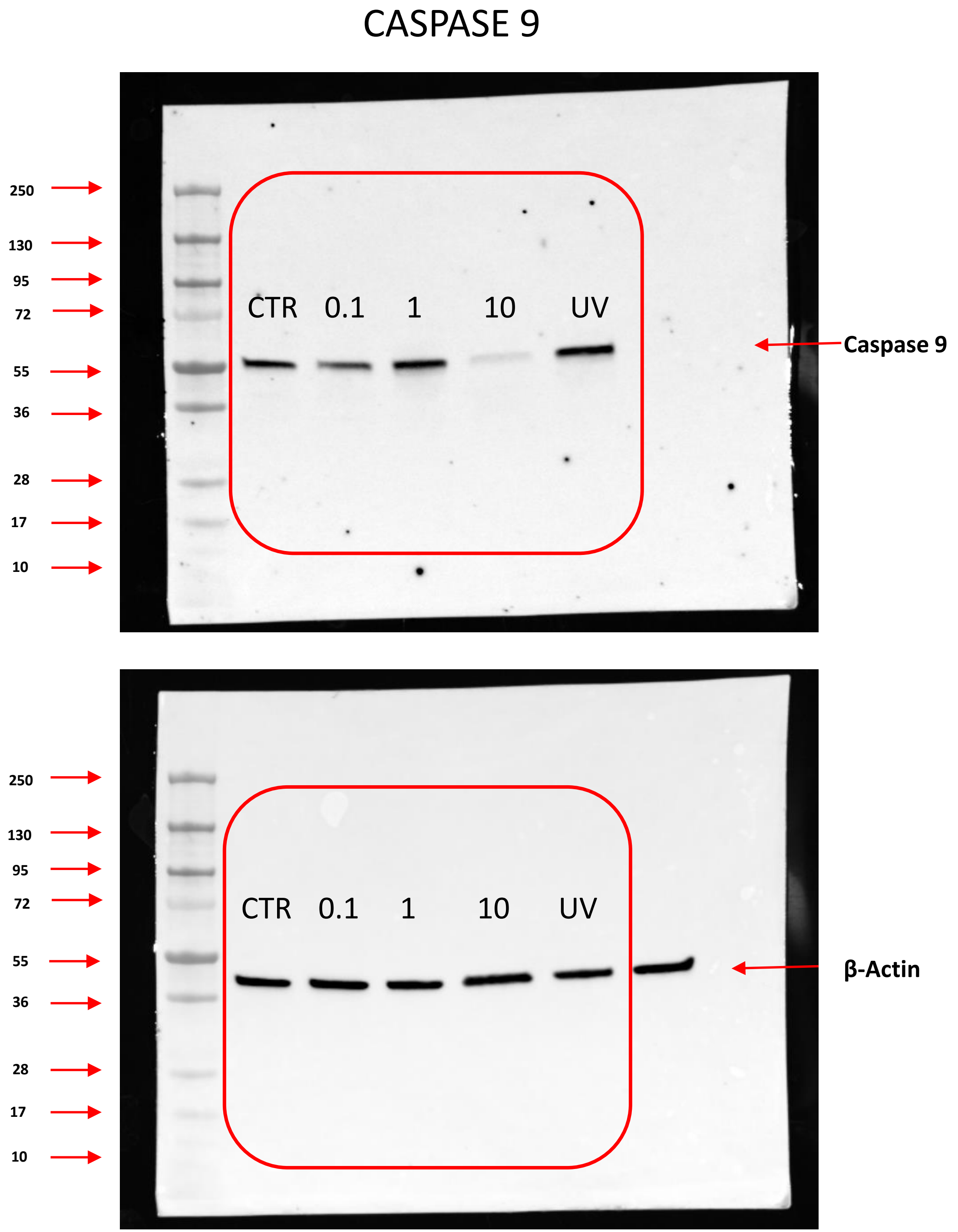
CASPASE 9
250
130
95
72
55
36
28
17
10
CTR 0.1 1 10 UV
Caspase 9
250
130
95
72
55
36
28
17
10
CTR 0.1 1 10 UV
β-Actin

FIG.6-7

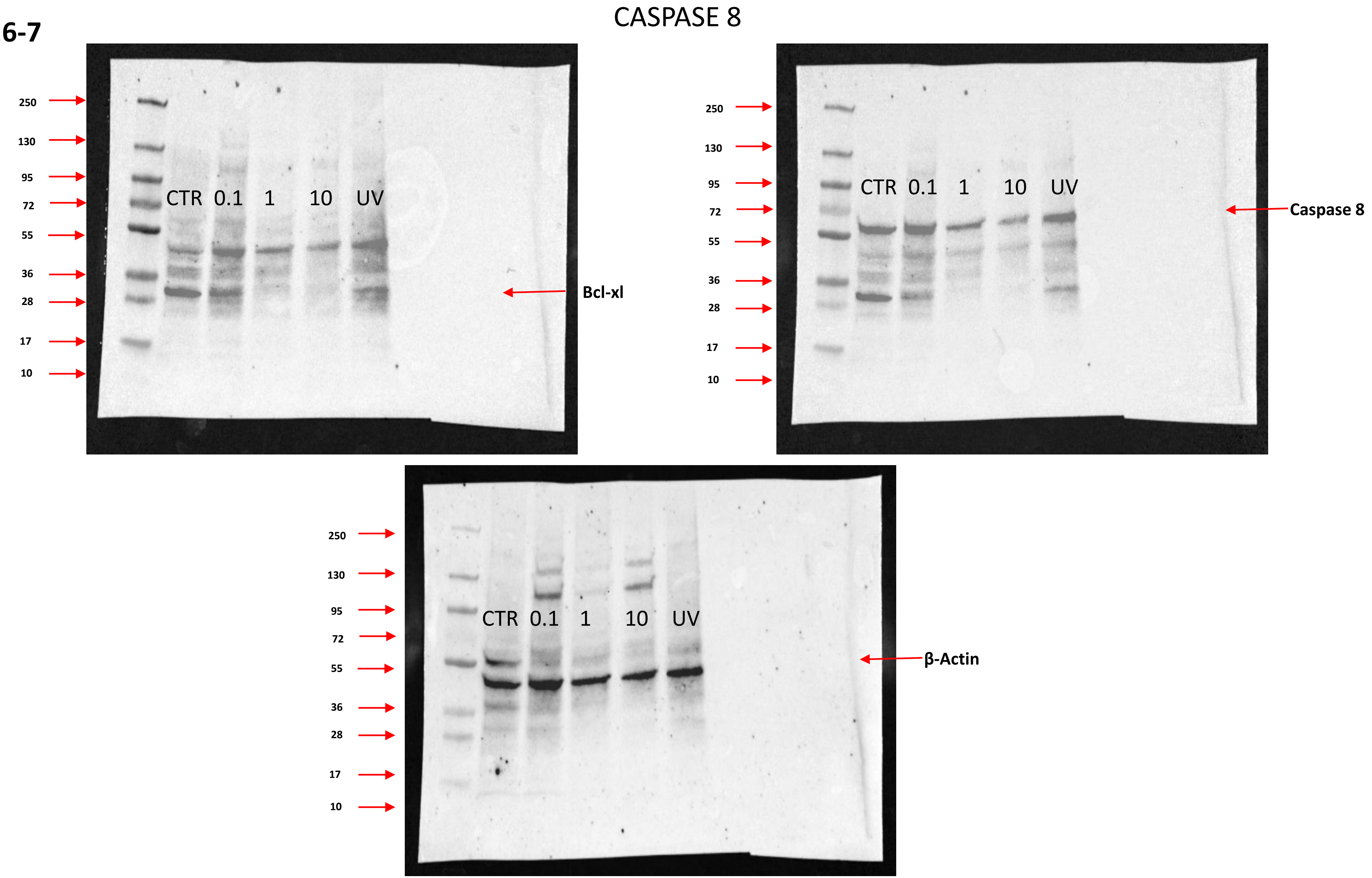
CASPASE 8
250
130
95
72
55
36
28
17
10
CTR
0.1
1
10
UV
Bcl-xl
Caspase 8
β-Actin

FIG.8

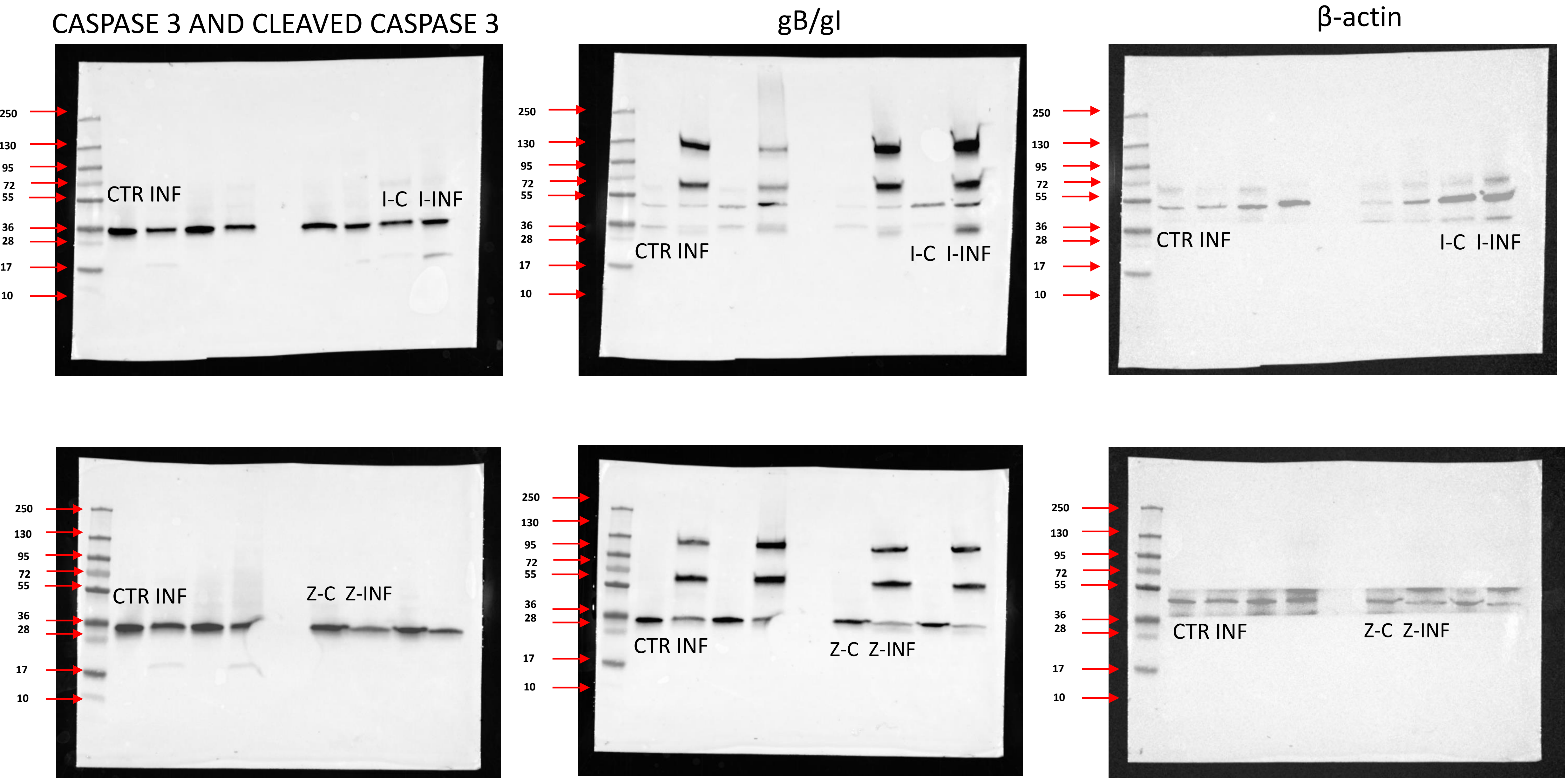

I-C and I-INF= Iomomycin control and infected cells; Z-C and Z-I= ZVAD control and infected cells

FIG.9

CASPASE 3 and CLEAVED CASPASE 3 DURING AUTOPHAGY INHIBITION AND INDUCTION

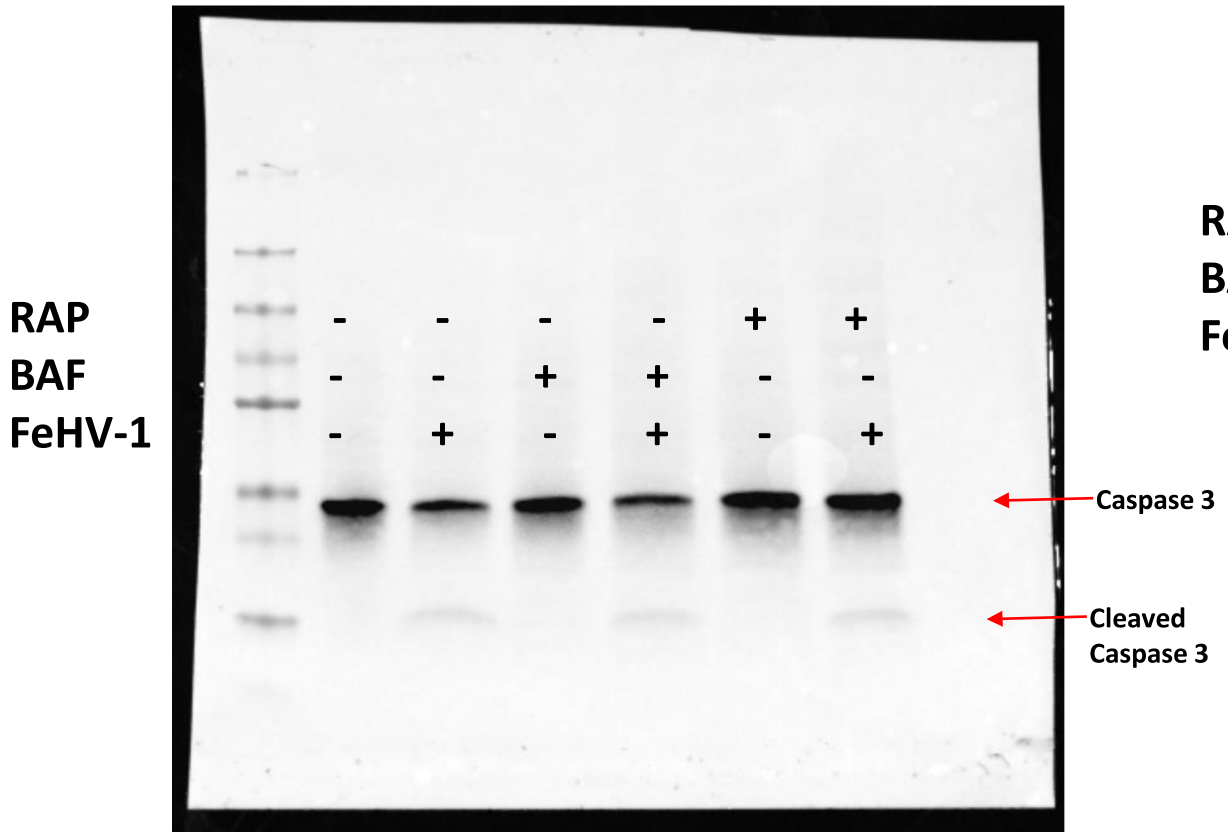

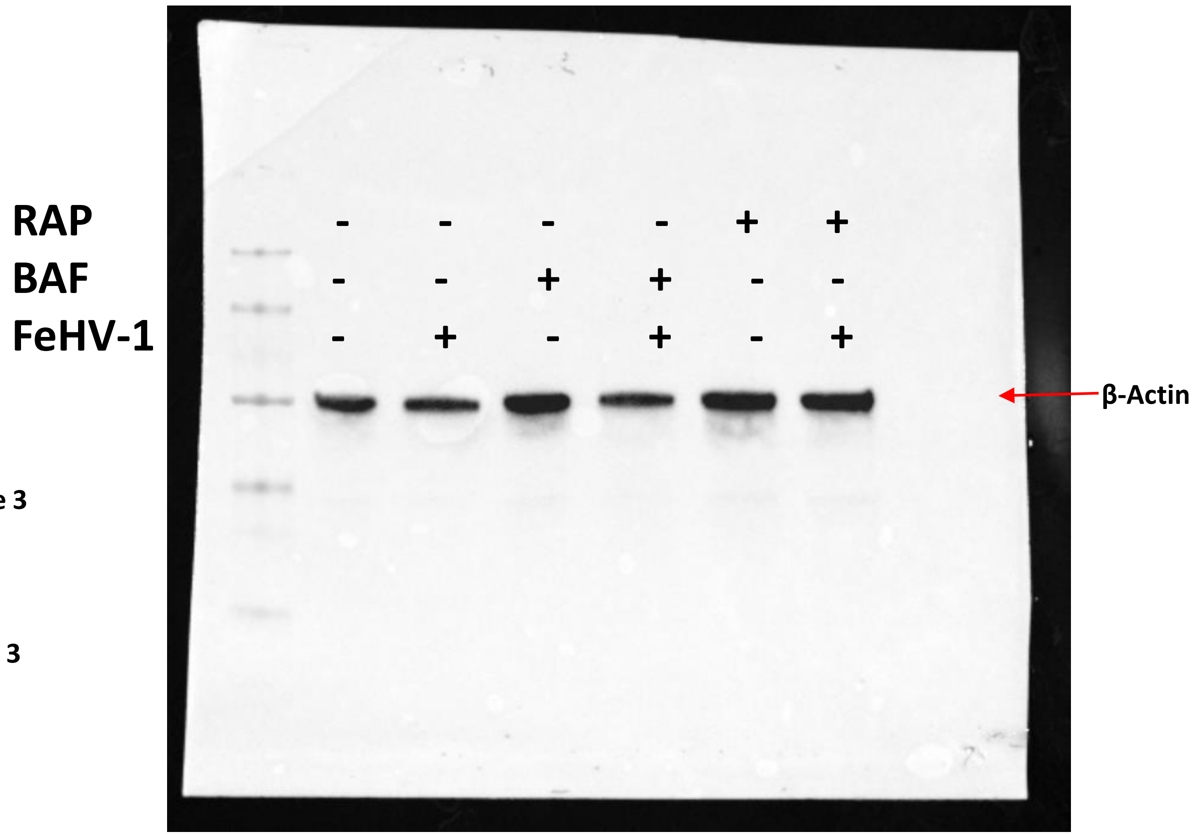

FIG.9

LC3 and p62 DURING Apoptotis INHIBITION AND INDUCTION

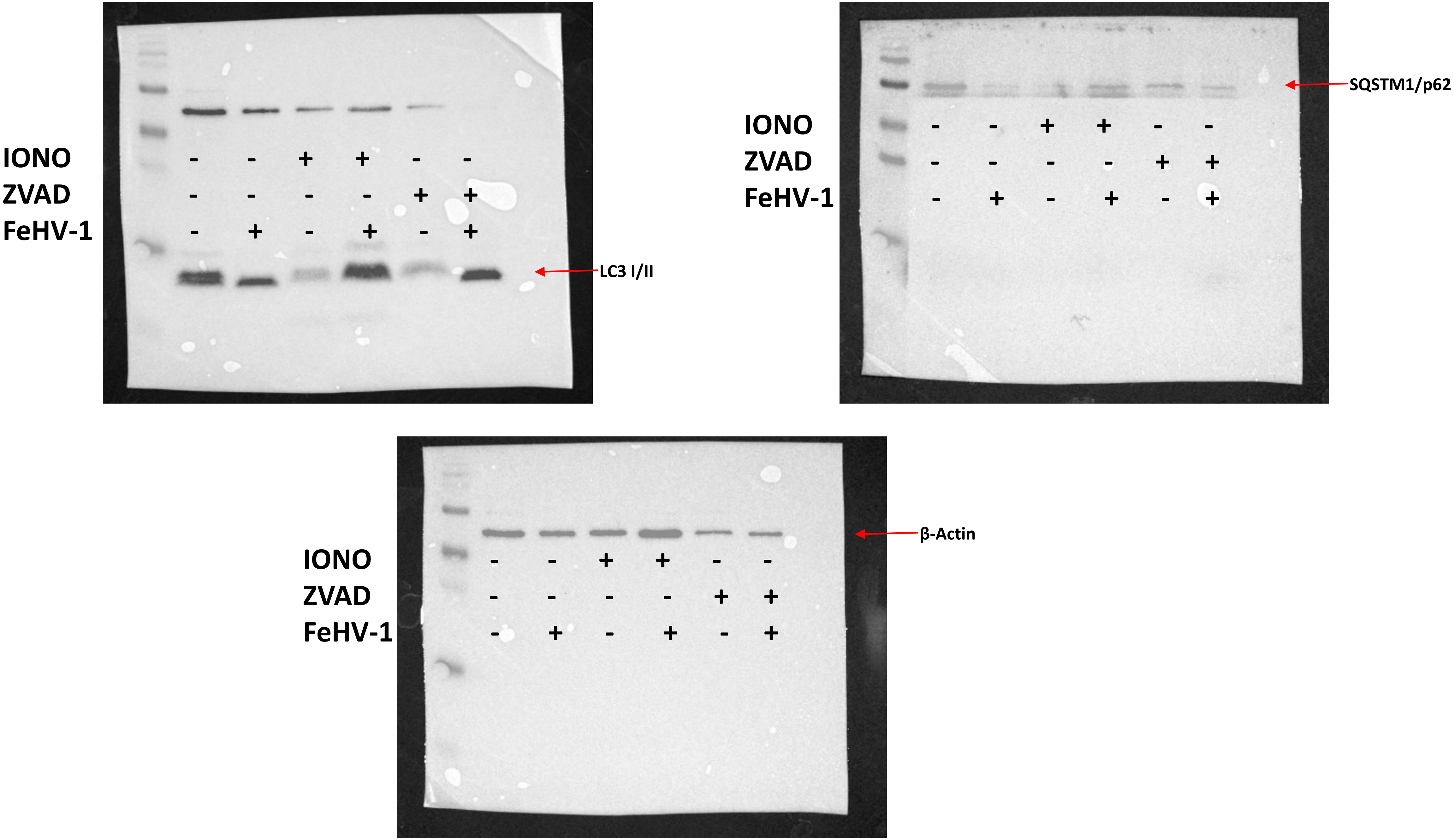

Supplement: Supplementary file 1 — Supplementary Material 1: Whole western blot membranes of figures 2, 3, 4, 6, 7, 8, and 9 [file 12985_2023_2267_MOESM1_ESM.pdf]
